# Supplementary material for: Vegetative cell wall protein OsGP1 regulates cell wall mediated soda saline-alkali stress in rice
Source: PeerJ. 2024 Feb 28;12:e16790. doi: 10.7717/peerj.16790 (PMC10908258; doi:10.7717/peerj.16790)
Supplement: Supplemental Information 1 [file peerj-12-16790-s001.docx]

| Primer | Sequence |
| --- | --- |
| OsGP1-F1 | 5’- ATGGCGTCATCGGCATTGCC -3’ |
| OsGP1-R2 | 5’- GCTCCGGCGAACGATCAGAC -3’ |
| OsGP1-F3 | 5’- AAAGCAGGCTCAGGGGATATCATGGCGTCATCGGCATTGCC -3’ |
| OsGP1-R4 | 5’- AGCTGGGTGCAGGGCGATATCGACGAGCTCGAGGCGGGCG -3’ |
| B5-R | 5’- ACCACCCCGGTGAACAGCTCCT -3’ |
| qOs18S-F | 5’- TTAGTTGGTGGAGCGATTTGT -3’ |
| qOs18S-R | 5’- GGCATTCCTCGTTGAAGACC -3’ |
| qOsGP1-F | 5’- CGAGGAGGACGACGACGATAAG -3’ |
| qOsGP1-R | 5’- ACCTGGACCGCCGGATGTT -3’ |
