# Supplementary figures and images for: Vegetative cell wall protein OsGP1 regulates cell wall mediated soda saline-alkali stress in rice
Source: PeerJ. 2024 Feb 28;12:e16790. doi: 10.7717/peerj.16790 (PMC10908258; doi:10.7717/peerj.16790)

**A****① BP reaction**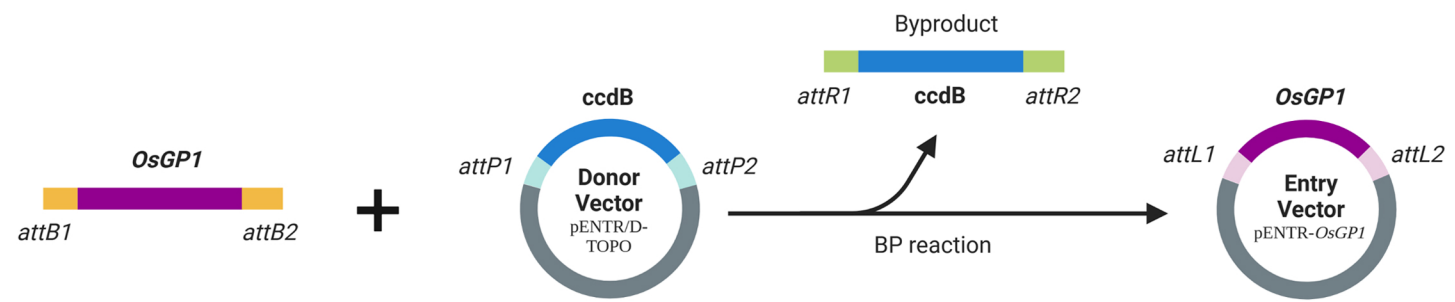**② LR reaction**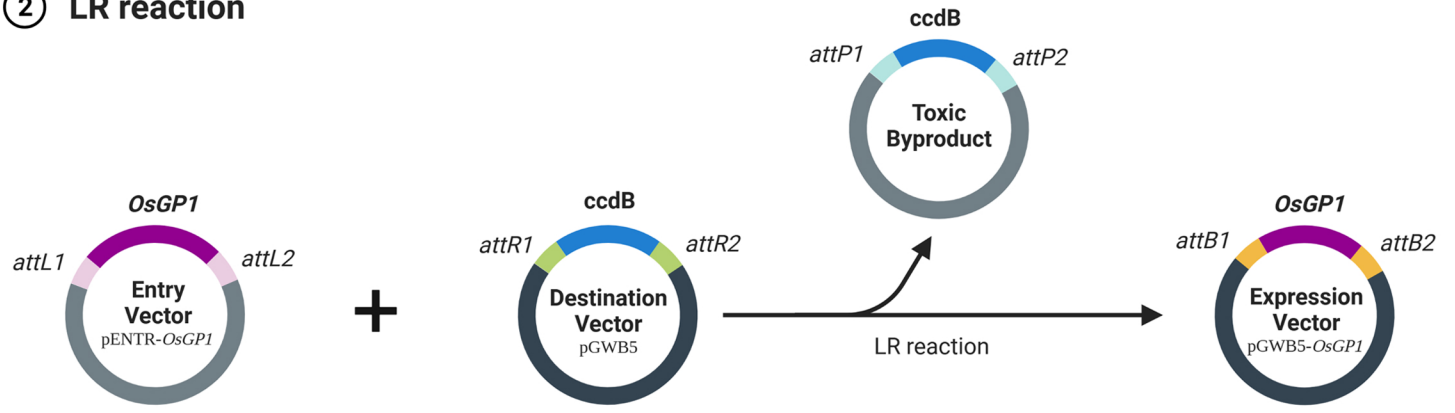**B**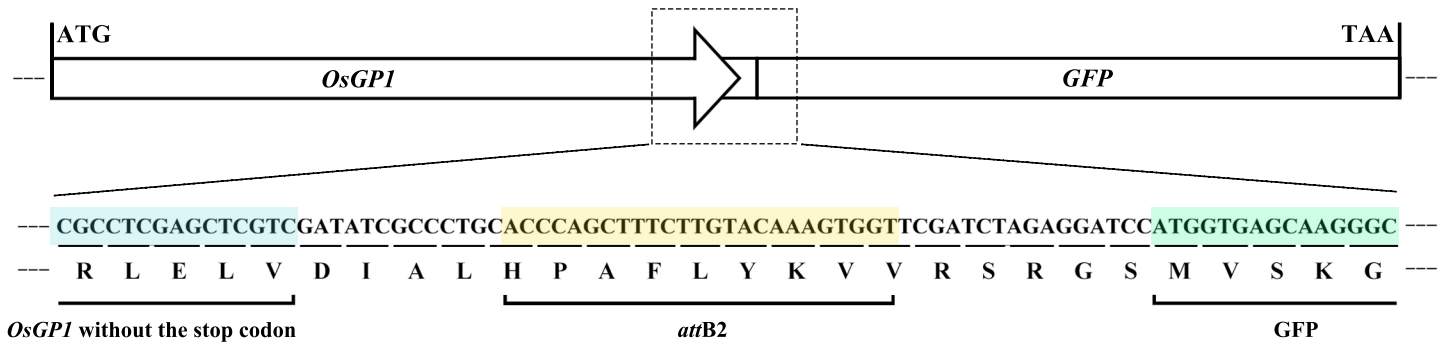

Supplement: Supplemental Information 2 — A, Gateway cloning technology used in this study. The construction strategy was created with Biorender (https://www.biorender.com/). B, Details of the linkage between OsGP1 and GFP proteins. [file peerj-12-16790-s002.pdf]
